# Developing fluency in a language of tactile communication

**DOI:** 10.3389/fresc.2022.1027344

**Published:** 2023-01-11

**Authors:** Neil Tuttle, Susan Hillier

**Affiliations:** ^1^ School of Health Sciences, College of Health and Medicine, University of Tasmania, Launceston, TAS, Australia; ^2^ Allied Health and Human Performance, University of South Australia, Adelaide, SA, Australia

**Keywords:** touch, physiotherapy, education, musculoskeletal, neurological

## Abstract

Touch has been an integral part of physiotherapeutic approaches since the inception of the profession. More recently, advances in the evidence-base for exercise prescription and “active” management have brought “touch” into question. This, in part, assumes that the patient or recipient simply passively receives the input rather than being an active partner in the interaction. In this article, we propose that touch can be used as a two-way conversation between therapist and client where each is engaged in tactile communication that has the potential to raise patient awareness and improve movement-based behaviour.

## 1. Introduction

“*Touch comes before sight, before speech. It is the first language and the last, and it always tells the truth*.” Margaret Atwood, The Blind Assassin 2000

Rather than delving into the physiology, this paper will focus on the practical aspects of assisting physiotherapy students and health professionals to become more fluent in a language of tactile communication when working with their clients or patients. We will apply a critical/expert lens by presenting narratives from our points of view as academic clinicians.

It has been suggested that “the same sensory, motor, and affective neural processes involved in our interaction with various concrete objects are activated when we understand, reason and talk about those objects” (1). We would suggest that the inverse is also true, where the same neural processes that are involved in verbal communication are also involved in tactile communication. This approach to physical communication is consistent with what is typically considered a biopsychosocial framework and perhaps even extends beyond the addition of the physical environment, as occurs in a biopsycho-ecological environmental model.(2) We will discuss how tactile communication has unique characteristics in the context of therapeutic relationships that may be overlooked or simplified. First, therefore, it may be useful to provide a brief theoretical context.

Interactions between individuals are often considered to be reason-based, where they focus on the intentions of one party, with the other party being a passive object who is either being interrogated or is a passive recipient of information. Such interactions can be seen as having a shared intentionality, which Chater et al. (3) define as “any cases in which people reach a commonly agreed interpretation, understanding, decision, or plan.”

Most of what is written about physical therapeutic interactions considers them to be reason-based—(1) the body-as-object, where the clinician is gathering information about another person, including whether a particular movement or touch is comfortable, (2) the body-as-subject where the clinician is perceiving information, or (3) an interaction as intentionality where the clinician is trying to communicate something such as reassurance, interest, or safety. In other words, the interactions are perceived as being active by the therapist and passive by the patient. It is just this perception of an active/passive dichotomy that has resulted in hands-on interventions being denigrated and considered to foster patient dependence.

Just like verbal interactions, physical interactions are perhaps better seen as interactive conversations with a shared intentionality. This concept has been elegantly extended by Øberg et al. (4) who describe an embodied-enactive interaction where “the dynamics of lived bodily engagement between physical therapist and patient contribute to and help to constitute the clinical reasoning process.” Sørvoll et al. (5) in another article in the same series provide an insightful discussion of an application of these concepts to paediatric physiotherapy.

Put another way, physical interactions, which are often considered to be reason-based, one-way communication, are in fact physical conversations between a therapist and patient and are perhaps more analogous to a dance (6) where one party ostensibly leads, but is often led by the other. This conversation is neither simply guiding or facilitating movement nor gathering or providing information. Rather, like any meaningful discussion, both parties come away with different perspectives. In verbal and, to some extent, non-verbal communication, the language is obvious. The structure, grammar, and language of tactile communication are not so obvious. Nor is it adequately taught to health professionals (7) and it is typically expected to be learned after qualification in clinical practice (8).

The remainder of this paper will provide a narrative from the authors' experience as clinicians and educators on both the language of tactile communication and on developing fluency in that language. Both authors have extensive experience as educators for entry-level and postgraduate clinicians. One author (SH) works primarily with people with neurological disorders, and the other author (NT) with people with musculoskeletal disorders. The paper will conclude with a synthesis and suggestions for improving the development of fluency in physiotherapy students and clinicians. We take the first person stance at times and have indicated the speaker with our initials, particularly when the perspective changes.

## 2. Neurological practice (SH)

In Neurological physiotherapy—as in other areas—there is something of a divide emerging between therapists: are we “hands' off” and work by prescribing exercise and coaching task-specific training with the intentions of fostering self-management or do we also touch our patients (clients) with the intention of maximising functional outcomes? Although the divide seems to be widening, I think it is a false dichotomy—there is no need to choose one exclusively over the other, nor do the two occur in isolation. Students are often trained in one “camp” as an undergraduate (depending on the school) and only on graduation appreciate there are other camps. The false dichotomy is not helped by group-based randomised control trials which are more suitable to evaluating standardised interventions while the heterogeneity of touch-based therapies makes them less amenable to this type of study design (9).

Of course, touch in a rehabilitation context can also be one-directional and reason-based such as communicating care or reassurance, or it can be procedural (assisting in manual handling). Some of the older therapeutic techniques, such as proprioceptive neuromuscular facilitation (PNF) and the original Bobath approach, also argued that touch could be facilitatory.

On the other hand, what about the use of touch in an educational way that promotes quality of performance in task-specific training? Is not that possible? Clinically, many of us do this; however, it is rarely, if ever, tested empirically. We do not know the evidence-base for this combination of “the dichotomy.” I would argue there is a biological rationale to justify investigation. That touch in a rehabilitation context is a form of highly accurate augmented feedback—first it invites attention to be directed to the somatic area of inquiry (for both parties) and then can provide questioning, confirmatory, additive or clarifying information in real time (as movement or physiological change occurs). This is the contested space, as touch has become associated with “passive” techniques. So it is important to understand that if feedback (augmented and otherwise) becomes an effective part of the perception-cognition-action loop then it results in improvement in feedforward—that is to say it up-regulates future behaviour. There is an implicit active process on the part of the client to process this exchange of feedback and to incorporate it into a change, hopefully for the better, which is in turn perceived and responded to by the therapist. If we take a systems perspective both parties are then engaged in a cycle of feedback and feedforward—both moving towards quality in performance and ultimately action.

So can touch become an active tool in movement re-education? Clearly I would argue yes. As neurological physiotherapists, we work with people who have (usually) a deeply impaired sense of self—physiologically, psychologically, and socially (10). The evidence for this is slowly emerging—the phenomenology of the “disabled body,” disrupted body schema and internal representations, reduced sense of agency and ownership, and impaired primary sensation all contribute. Intuitively, it makes sense that a predominantly non-cognitive process of body awareness is difficult to remediate using cognitive means. Rather, because this awareness is based on primary sensations of predominantly the kinaesthetic senses, doesn’t it “make sense” that touch becomes the instructional medium of choice?

So how does this manifest in a rehabilitation setting? It is well known that feedback can be intrinsic (generated internally) or extrinsic (from external sources). Touch from a therapist can link these sources. The therapist's hands can provide tactual information that is perceived by the client as intrinsic information—regarding where they are in space, where one part is in relation to another and how they are moving (speed and direction). This augmented proprioceptive feedback (if you like) arguably updates the individual's body schema and allows more accurate feedforward in the classic motor learning loop. That the feedback occurs in real time means it is a form of “knowledge of performance.”

I suggest this requires a distinction between touch as assistive (that is offering augmentation to the actual movement ranging from passively moving the client through to being partly or minimally assistive) compared with touch that is resistive (that offers a resistance to the client's active movement to increase recruitment). These then can be compared with a third alternative—touch that is neither physically assistive or resistive but is questioning and/or informative tactually and that the client can be directed to pay attention to this inquiry as a focus for improvement in perception-cognition-action. If we stay true to motor learning principles though, such augmented or extrinsically derived feedback will be gradually withdrawn in favour of intrinsic-only feedback to enable self-agency (i.e., avoid dependence on the therapist as the source of feedback).

We are particularly interested in this conversational tactile language—where suggestions, questions, responses, and acknowledgement of change in a process using tactual inquiry can be implicit or explicit. This can be a mutual exploration of the person's internal state that allows the tracking of change. Tactual engagement may allow an interaction that is somatically more congruent than what can occur with verbal communication.

## 3. Musculoskeletal practice (NT)

The criticism of hands-on therapies as fostering dependence is perhaps even stronger in musculoskeletal practice than in the other areas.^1^ Similar to the neurological setting, touch in a musculoskeletal context can be a one-directional, reason-based interaction such as gathering information (swelling, temperature, or resistance to movement), communicating (caring or reassurance), or having a physiological therapeutic effect. Even when physical interactions are considered to be two way, it is most often described as simply being a question of whether what is being done is comfortable or acceptable. So how can something like massage or what is referred to as passive mobilisation be considered to be an active conversation with shared intentionality or even embodied enaction involving both parties?

It is common practice for musculoskeletal therapists to interleave therapeutic and information gathering interactions by reassessing a patient's movement or function multiple times within a treatment session. I have asked a number of therapists whether there are times when they know that a person has improved before they reassess, and if so, how do they know? The answer is invariably yes; they do know at least some of the time, and they know because they, as the therapist, feel a change during the treatment application. Although in this instance there are two reason-based interactions occurring; one intentional of having a therapeutic effect and the other of gathering information; this may still not qualify as a two-way conversation.

When I ask the follow-up question of whether they adjust what they do to maximise the change they perceive as being related to patient improvement, the answers become more diverse including

*No, [I was taught that, or I think that] I need to maintain a consistent technique to ensure a consistent dosage*.

*I suppose so, but I’ve never really thought about it*.

*Or less frequently, Yes, I continually adjust what I do to maximise the effect*.

Regardless of whether the therapist responds consciously or unconsciously to the patient's response, it is at this point that the physical communication can be considered a two-way interaction. It may still, however, be reason-based interactions with the therapist simultaneously gathering information and having a therapeutic intention.

Most therapists and patients share a common intention or goal—wanting the patient to improve. So there is a shared intentionality. The patient's and the therapist's perceptions, however, may be that the patient will just lie there passively and be treated. So, although there is a shared intention (goal), this may still be seen as a one-way process where the patient either receives input from or provides information to the therapist. Similarly, a student sitting in a lecture given by an attentive lecturer who responds to the non-verbal responses of individual students in the room could still be considered a passive recipient in spite of both parties having the intention of increasing the student's learning. O’Madagain and Tomasello (11) expand the idea of shared intentionality to include that it enables people “to do things that no one of them could do alone.”

What might these conversations with shared intentionality or embodied enaction between therapist and patient look like in musculoskeletal physiotherapy? I would suggest that it might look exactly like what would occur if the patient was a passive recipient. The activity of a student in a lecture described above would be undetectable in an audio recording of the lecture because the medium of the interaction was not perceived. Similarly, the activity of a patient is likely to be undetectable by someone watching the interaction because the medium is either not visible or not noticed.

Let us return to therapist–patient interactions. An embodied action is difficult to reduce to words, but an imperfect translation of such a conversation lasting several minutes might be something like in the box below.

Verbal conversations can occur as entirely verbal interactions, as with a phone call, or the verbal component can be augmented by non-verbal components such as gesture or touch. Similarly, tactile conversations can occur as completely tactile interactions or be augmented with a verbal component. Below is a hypothetical example of such a conversation pieced together from personal observations and discussions with both therapists and patients about their experience during so-called passive mobilisation of the cervical spine. Although the verbal content shown here can augment the conversation, we would suggest that an entire tactile therapeutic conversation can occur without any verbal interaction.

In the following table:

Tactile content from the patient is in plain text;

*Tactile content from the therapist is in italics;*

**Verbal content from the patient is in bold;**

***Verbal content from the therapist is in bold italics.***

**Table:** 

*Therapist*	Patient
*Let*'s *see what movements are preventing you from achieving your functional goals? I suspect here is probably OK.*	Doesn't evoke a protective response.
*It may have something to do with what is happening here. Hm, interesting, (feeling limitation of segmental* *mobility) I suspect this may be part of the story.*	Some protective reaction.
***What do you feel when I do this?***	**Yes, I can feel that a bit**
*I wonder what happens here? Is it the same as this on the other side [directing and focussing attention]? No, this is different.*	Different muscular and movement response to the two movements
***How is this different from this?***	**I can feel the second one more**
***Which one is more similar to your symptoms?***	**The second one**
*Continues to explore symptomatic location/s varying directions, with differing forces, etc. Partly to gather information and partly also to focus patient's attention and increase the acuity of their perception*	Yes, there seems to be a lot going on there. I don’t know what you are doing differently, but there are different symptom and muscular responses as you change what you are doing. I’m surprised at how much my response changes when you do something that is very slightly different.
***I’m looking for how well these individual vertebrae move. For example, what happens when I do this?***	**I can feel that**
***Besides the pain can you feel any difference in the movement there?***	Hm, I think it's different, hard to notice anything besides it being uncomfortable. **I don’t know, can you do it again?**
***So how is this—*** *Performs an analogous movement **compared to this?** Performs the more symptomatic movement*	Oh, that's what he means by movement. **Yes, that one feels tighter.**
*I'm feeling some protective responses happening. It could be useful for the patient to have a sense of those responses to gain some control over them. I’m feeling increased movement and their protective response decreasing and that by adjusting the direction, force, rhythm, etc., I can increase the rate of improvement (* *12* *).*	** **
***As I continue doing this what is happening to that tightness (discomfort)?***	Probably only notices when therapist draws their attention back to the area. **I think it is getting a little easier.**
***Yes, good. It feels like it is loosening to me too. My intention is to increase that mobility which as we’ve said is probably related to your symptoms***	As the treatment continues may notice other changes happening. Protective responses persisting after they were “necessary”, a sigh, a relaxation of broader holding patterns …
*So this can move in a way that is safe and comfortable. Let*'s *see how we can expand that movement [gradual changes and adjustments] to include a larger range, other directions and/or movements of other structures.*	Yes, that's still OK, no, not that, yes, no, hm, I think so, yes, OK, I think that's getting easier now. Yes that's not so bad now.
***Good, that***'s ***moving easier there, so what has changed*** *[maybe exploring and comparing some other areas]?*	**I don’t know, maybe I can just let go of it more because I know that it won’t always hurt me.**
*Yes, and you found a way that you could move the other one, so let’s see what we can find here.* *So what about here?*	*That* feels kind of like the other one did at the beginning.
*Now that these are easier, let*'s *sit you up and see what turning your head is like now compared to before.* ***You remember how far you moved before? Can you picture that movement? Can you imagine moving further or differently now?***	Attention to quality of movement. Facilitate translation of response that occurred with “passive” movement to what occurs with active movement.

Mechanisms underlying the development of musculoskeletal pain, and therefore what needs to be affected by effective treatment, are far beyond the scope of this paper. What is agreed upon by most physiotherapists is that in addition to the possible physiological effects of manual therapy, the way a person moves forms part of the story. I would suggest that much of the tactile conversation that occurs during a manual therapy session is about how the therapist and patient use themselves and the concepts and attitudes (e.g., fear avoidance or responses to pain or effort) that underlie that usage.

Further conversations might explore how what the patient learned in that tactile conversation can be generalised. When questioned, some patients have described how, when they noticed symptoms at some point after a treatment session, they were reminded of differences in the quality of movement that had occurred during the treatment session and were able to find that quality again. For example, “I noticed my shoulder being up when I was driving and remembered how it felt here.” In this instance, nothing was mentioned in the physiotherapy session about the height of their shoulder. Questions can then arise and be addressed either tactilely or verbally as to whether this is part of a broader pattern that includes other areas and/or movements.

In neurological practice, the interplay of the tactile and verbal conversation is perhaps even more clear. I (SH) encourage my clients to describe to me the sensory experience of their impairments, and we use touch to elucidate, particularly “when words fail.” Using touch as a conversation, we can explicitly explore the lived experience of spasticity, weakness, or paraesthesia, and movement as a sensed behaviour. For example, “when I do this (input to facilitate, inhibit, or inform), what happens to how you move?” As a therapist, we can ask a question by creating a kind of puzzle. Again, this fits with Dynamic Action Theories of motor control—where movement puzzles can be explored and solutions generated. What we are suggesting is that inherent in this process is sensory experience (in both feedback and feedforward) as well as enhanced perceptual learning to explore and understand different states. This is illustrated where we may use touch to encourage weight shift during stance in a certain direction, which can be accompanied by a sensory and verbal exchange of how one knows where one is, “how do I know I have my weight on my left leg?”, “where do I feel more stable”, “how do I know I am more stable in this position”?

## 4. Learning and teaching

As mentioned at the beginning of this article, the skills of physical communication are largely ignored in physiotherapy education (7) and are typically expected to be learned through clinical practice (8). One exception to this is Geri et al (13) who advocate strongly for the need for knowledge and skill development in the manual therapy component of physiotherapy education. Even this, however, seems to view the therapist as the actor providing an input to the patient.

How then do we assist our students learn to use touch in a way that affords embodied enaction? Norris and Wainwright (14) describe three stages in which students develop skills in touch: from uncertainty through emerging familiarity to realities of touch. Although they discuss the embodied nature of touch, discussions of interactions are largely limited to the reason-based interactions described above, and the communication described is predominantly about what the therapist communicates to their patient. What we are attempting to describe goes beyond this one-directional communication and is perhaps more analogous to learning a verbal language.

Early theories of first (verbal) language acquisition have moved beyond either a process of reward-based operant conditioning or the growth of an inherent language module to a recognition that infants use more complex strategies than were previously thought possible (15). Importantly, language is not acquired completely passively. Parents universally and seemingly automatically adopt strategies, including the pace and pitch of speech, repetition, and presenting new words in a variety of contexts, that have been shown to effectively facilitate language acquisition. We know of no research that has investigated the development of tactile language acquisition in infants. It can, however, be expected that because we and all our students have been touched throughout our development and engage in tactile communication in a range of non-therapeutic settings, we all have some level of tactile language. We think that it is also reasonable to assume that this language includes the type of non-reason-based interactions we previously referred to as embodied action. It would be interesting to consider what “universal and seemingly automatic” strategies parents adopt to assist children in the development of their local tactile language.

Because we all have developed some form of tactile language, learning therapeutic embodied enaction is more analogous to learning a second language. Perhaps the concept of second language acquisition that is most relevant to our discussion is “a holistic and reflective approach which considers the learner as a whole, with his or her emotions, empathy, personality, and identity, and has an essential role to play in language learning to enhance student engagement and success.” (16)

So, how can we help our students learn this second (tactile) language?

First, it is necessary for students to recognise that two-way physical conversations are possible and then for them to have sufficient body awareness to detect themselves and later their clients accurately in space and time.

For several years, I (NT) was fortunate to lead the first practical session for commencing physiotherapy students. I saw this as an opportunity to set the stage, and although not initially conceptualising it as such, to enable students to experience a quite simple version of the type of embodied enaction that is being discussed here. The session started by doing a simple movement, such as turning their head side to side. By then mimicking the movement of a few of their colleagues, they recognised that there was a wide range of strategies to turn one's head—all of them normal. Students then went through a series of activities where they turned the heads of their colleagues and had theirs turned. By the end of a couple of hours they knew from their own experience of being guided only by the physical interaction that they could find ways of moving someone else that were both easier for themselves and more comfortable for their partner.

Any conversation requires the ability to receive^2^ an input and express an output. To fluently converse in a language of physical communication, students require training in a language of somatic experience. In order to be able to read and write written text, one needs to have adequate visual discrimination. So ensuring the ability to perceive the necessary information becomes the first training need—how do we develop our own exact sense of self in order to offer the clearest tactual conversation to our clients with impairment. It is often presumed that all students have comparable levels of perception. Some of my unpublished research indicates that is not the case, with individual students' ability to detect differences or changes in stiffness varying by at least a factor of four.

The process of refining perception and usage is taken much further in training to become practitioners in the Feldenkrais method, where the first year (and more) is spent explicitly training the sense of self through “awareness through movement” lessons. In learning a language, other professions place a similar emphasis on self-development first. Wine-makers train their sensory perceptions for taste and olfaction systematically prior to making-wine. Surgeons implicitly train in sensory appreciation ready for delicate surgery where vision is often not available as a feedback sense for accuracy—haptic simulation is being developed so that they can experience accurate sensory feedback for example during throat surgery they “encounter” accurate changes in soft tissue density in the simulation model. Perhaps it is time we spent more time with physiotherapy students to improve their own sensory discrimination, acuity, and body awareness—a focus on the self as well as on the other?

It is perhaps only after there is an acceptance of the existence of embodied enaction and sufficient sensitivity to perceive it that the grammar^3^ and then a language can be developed.

Similar to what is described in the literature, I (NT) only began to develop fluency in embodied interaction after years of clinical experience in musculoskeletal physiotherapy. Perhaps this is because of how I, and I am sure many others, were taught. A technique would be demonstrated, and we would then try to duplicate it. The instructors would watch what we did, and maybe we exchanged movement diagrams of what we felt. Once we had some skill, I remember Geoff Maitland saying that we needed to “get inside the joint.” I do not however recall him or others ever saying what one does once we got there. At the time, I did not think that idea needed any further development. It now seems to me that trying to guide the development of physical skills using only visual and verbal input will mean that if a fluency in embodied enaction develops at all, it will only occur later in one's career while being immersed in the physical conversations (e.g., clinical practice).

Rather than waiting for perhaps years of clinical practice, how can we start to assist students and novice therapists to develop these skills? Certainly demonstrating, watching, and practicing skills is important. It is also useful for us to feel students practicing on us and for us to demonstrate on them. Even then, however, there is a step between what one feels under someone else's hands and what they do with their own hands. This is perhaps analogous to learning a language by listening and speaking separately without necessarily having conversations. We propose a further step where the interaction between educator and student is able to amplify the process and is analogous to the immersion used in teaching a second language.

Because this therapeutic tactile language is not a native language to either of the participants and the native tactile language of each party is different, the strategies used in a “Lingua Franca” conversation (where the language is not native to either speaker) become relevant. These strategies have been described as the “pragmatic moves participants enact in the process of meaning co-construction and negotiation, which can be seen as a complex set of pragmatic moves aimed at ensuring mutual understanding, where negotiation of meaning is jointly coconstructed by the participants in the communicative event.” (17) In other words, the authors suggest that communicators work together in creative and often unpredictable ways to convey and receive information. It is therefore important for educators to facilitate learners' skills in finding strategies to ease a mutual construction of meaning.

**Figure 1:**
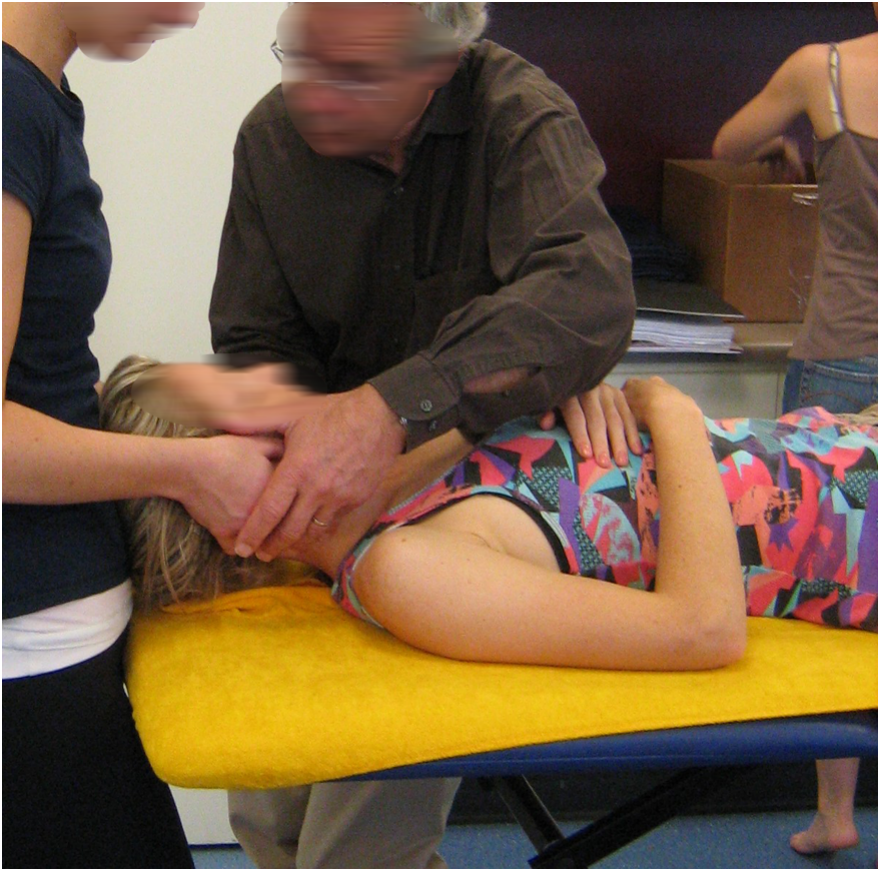
Examples of an instructor aiding learning of embodied enaction. Image at right courtesy of Dr. Steve Obst.

**Figure 2:**
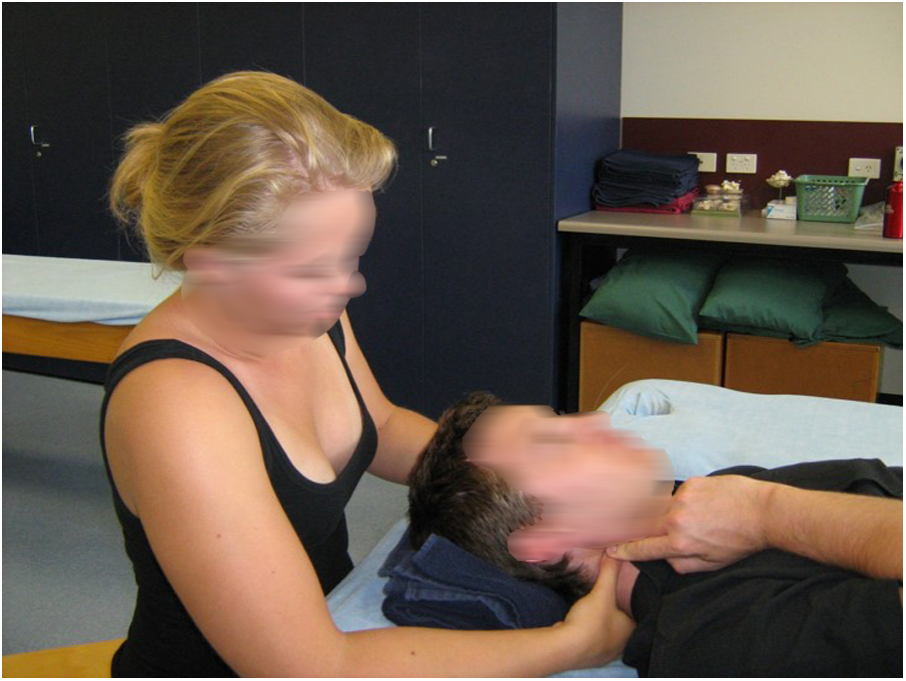


It seems intuitively obvious that fluency in the language of physical interactions is best developed through physical interactions and that this can be learned most effectively in the first instance by interactions where the instructor can physically guide the student during interactions. Figures 1 and 2 may look like teaching “how to” do techniques. Rather, they are images of the instructor clarifying what is being felt and how to interpret it, and more importantly, guiding the student's response to the changes occurring in real time simultaneously under both of their hands. The perceptions are direct, without an intermediary of vision or description.

In neurological practice, informative, inquiring, and enabling touch can be similarly demonstrated to students, however, in clinical practice, the heterogeneity of perception on the client's side is often manifest. How can we, in a sense, enter the experience of the person with a stroke or multiple sclerosis? Given the individuality inherent in such a phenomenological approach, we can encourage the student to approach each client from the stance of a beginner with the opening implicit questions “how do you feel” (literally), “what is this like?” and “what do you experience as your arm moves?” This requires the student to take on the role of an explorer rather than an expert. It leads more to discovery learning on both sides compared with guidance. We acknowledge that this mind-set is hard for students who are under pressure to adopt expertise to pass technique-based exams. However, we would argue that the current focus on competencies and practise standards allows us to move our authentic assessment more towards a state of being with our clients rather than a dispenser of techniques.

## 5. Summary

In this critical view, we propose not only that touch is an effective therapeutic tool, but that it is far from being a passive modality. Rather, it involves interactions and conversations that can “touch” aspects of both our lives and our patient's lives beyond what is possible verbally. Educating our students in the language of touch will require discourses such as those we hopefully have initiated in this article—to re-consider tactile therapies as more than passive techniques, to find ways to communicate the language of embodied interactions in more diverse and subtle ways, and to elucidate the teachable “dance-steps” to students in a way that can be adopted in our curriculum-heavy undergraduate and postgraduate programmes.

## Footnotes

^1^Interestingly this same criticism does not seem to be levelled at other interventions used in musculoskeletal practice such as medication, injections, or surgery where, I would argue, the patient is truly passive.

^2^It is intentional that this is described as to “receive” an input used here rather than “perceive” an input. We would suggest that the interactions can still occur when working with people with neurological conditions that result in a highly impaired or even absent ability to perceive physical input.

^3^Grammar is defined in the Oxford Dictionary of English Grammar as: The system of a language, traditionally encompassing syntax (the structure of sentences) and morphology (the structure of words).
